# Edge-based network analysis reveals frequency-specific network dynamics in aberrant anxiogenic processing in rats

**DOI:** 10.1162/netn_a_00251

**Published:** 2022-07-01

**Authors:** Yin-Shing Lam, Xiu-Xiu Liu, Ya Ke, Wing-Ho Yung

**Affiliations:** School of Biomedical Sciences, Faculty of Medicine, The Chinese University of Hong Kong, Hong Kong; Gerald Choa Neuroscience Centre, The Chinese University of Hong Kong, Hong Kong

**Keywords:** Edge-based network analysis, Anxiety, Phase transfer entropy, Phase locking value, Theta oscillation

## Abstract

Uncovering interactions between edges of brain networks can reveal the organizational principle of the networks and also their dysregulations underlying aberrant behaviours such as in neuropsychiatric diseases. In this study, we looked into the applicability of edge-based network analysis in uncovering possible network mechanisms of aberrant anxiogenic processing. Utilizing a rat model of prodromal Parkinson’s disease we examined how a dorsomedial striatum–tied associative network (DSAN) may mediate context-based anxiogenic behaviour. Following dopamine depletion in the dorsomedial striatum, an exaggerated bottom-up signalling (posterior parietal-hippocampal-retrosplenial to anterior prefrontal-cingulate-amygdala regions) and gradient specific to the theta frequency in this network was observed. This change was accompanied by increased anxiety behaviour of the animals. By employing an edge-based approach in correlating informational flow (phase transfer entropy) with functional connectivity of all edges of this network, we further explore how the abnormal bottom-up signalling might be explained by alterations to the informational flow-connectivity motifs in the network. Our results demonstrate usage of edge-based network analysis in revealing concurrent informational processing and functional organization dynamics across multiple pathways in a brain network. This approach in unveiling network abnormalities and its impact on behavioural outcomes would be useful in probing the network basis of neuropsychiatric conditions.

## Author Summary

This study employs a unique approach to study the neural basis of anxiety by quantifying the correlation of concurrent fluctuations in information flow and connectivity between all edges of the brain network involved. This is the first time that such an edge-based technique is used to analyse field potential signals of the brain in an animal model of a neuropsychiatric disorder. We demonstrate how such edge-based analysis complements other analyses such as local field potential power and gross network changes. This work helps lay the foundation for future investigations into decoding the network aberrations of a wide spectrum of neuropsychiatric disorders.

## INTRODUCTION

Analysis of brain functional connectivity at the network level in both health and disease is a rapidly growing field (Bassett & Bullmore, 2009). However, current research lacks extensive interrogation into the complex network coordination across multiple pathways, resulting in incomplete understanding of physiological, psychiatric, and emotional disorders. Elucidation of the interaction between the connections between two component regions, or edges, across the entire network can reveal more extensive information on the functional coordination between sets of pathways. Network configuration is well recognized to be nonstatic and exhibits strong temporal fluctuations and functionally relevant patterns (Calhoun et al., 2014; Faskowitz et al., 2020; Müller et al., 2020; Shine et al., 2015). Thus, to truly unveil network-wide coordination of complex cognitive processes, it is crucial to understand the relationship between network edges via quantifying the temporal fluctuations in their properties. Interactions between brain regions occur in a frequency-specific manner via oscillating field potentials (Buzsáki et al., 2013). In recent years, phase-dependent measures of brain oscillations used as network parameters offer distinct advantages in quantifying functional and information flow relationships between brain regions, primarily due to their independence on the amplitudes of the signal (Robinson et al., 2013; Siems & Siegel, 2020). While phase locking value (PLV) is a measure of functional connectivity between two regions (Aydore et al., 2013), directed phase transfer entropy (dPTE) can reveal directional information flow or transfer between them (Lobier et al., 2014). Such metrics allow the interaction between network edges to be robustly quantified and compared.

Anxiety disorders constitutes the most common type of psychiatric disorder (Bandelow & Michaelis, 2015). Anxiety is an innate emotional state induced by potentially threatening stimuli exhibited by almost all animals possessing higher cognitive capabilities (Steimer, 2002). It involves multicircuit brain-wide integration of environmental information from the sensory cortices and the internal psychological state of the animal from higher cognitive centres of the brain (Barbot & Carrasco, 2018; Martin et al., 2009; Sussman et al., 2016), allowing individuals to respond to environmental threats and uncertainties in accordance with internal goals and motivation appropriately. In rodents, fMRI studies have shown the recruitment of multiple associative brain regions in anxious and introspective states (Becerra et al., 2011). Fear and anxiety are also associated with multiple groups of neural pathways across associative regions (LeDoux, 2000). Theta band brain activity alterations of different brain regions such as the hippocampal and frontal regions in anxious states are also well documented (Buzsáki, 2004; Cavanagh & Frank, 2014; Jacinto et al., 2013; Soltani Zangbar et al., 2020).

From comprehensive tracing and functional studies, the dorsomedial striatum (DMS) exhibits both close functional connectivity and receives broad excitatory projections from associative brain regions encompassing the frontal cortex, cingulate cortex, posterior cingulate cortex, parietal associative cortex, and amygdala and hippocampus (Delcasso et al., 2014; Greene et al., 2019; Hunnicutt et al., 2016). Such regions are recruited in brain processes such as environmental context processing, memory retrieval, and social interaction (Cwik et al., 2014; Delcasso et al., 2014; Glimcher & Fehr, 2014; Hunnicutt et al., 2016; Ritchey & Cooper, 2020). Studies have demonstrated that manipulating interactions between the medial prefrontal regions and the DMS can induce avoidance and approach behaviours of rodents (Gunaydin et al., 2017; Loewke et al., 2020). Therefore, the striatum and closely associated cortical and subcortical brain regions may constitute a network that bridge contextual information and behavioural output, which in turn regulates the expression of anxiety. Specifically, we hypothesize that a network consisting of associative cortical regions demonstrated to have significant projections and close functional associations to the DMS in rats (Delcasso et al., 2014; Genzel, 2020; Hunnicutt et al., 2016; Navarro-Lobato & Genzel, 2020) is under the regulation of the DMS dopaminergic innervation and is critical in the genesis and expression of anxiety. These areas include the medial prefrontal cortex (mPFC), the anterior cingulate cortex (ACC), the central nucleus of amygdala (CeA), and components of the default mode network posterior medial subnetwork encompassing the retrosplenial cortex (RSC), the parietal associative cortex (PtA), and the dorsal hippocampus (dHip).

The function of the striatum is tightly regulated by dopamine, a key mediator in a multitude of higher ordered cognitive and emotional states, such as motivation, cognitive flexibility, and mood regulation (Cavanagh et al., 2017; Seo et al., 2008; Westbrook & Braver, 2016). The projection from the substantia nigra to the striatum, the nigrostriatal pathway, is the largest dopaminergic pathway in the mammalian brain. DMS has been demonstrated to directly influence avoidance behaviours and anxiety in both rodents and humans via dopaminergic signalling (Hilbert et al., 2015; Nguyen et al., 2019); In disorders of defunct dopaminergic signalling such as Parkinson’s disease (PD), although motor symptoms are the major manifestations of the disease, anxiety is extremely prevalent during the prodromal phase of the disease. Anxiety and depression are also regarded as preclinical risk factors in PD development (Chen & Marsh, 2014; Meireles & Massano, 2012; Pontone et al., 2009). Therefore, the loss of dopaminergic signalling in nonmotor territories of the striatum could be a precipitating trigger in the development of anxiety (Erro et al., 2012).

Here, to investigate the network basis of anxiety induced by an artificial depletion of dopaminergic innervation of the DMS via direct 6-hydroxydopamine (6-OHDA) injection (Alvarez-Fischer et al., 2008), we propose an analysis framework, similar in concept to cofluctuations of connectivity patterns demonstrated recently by Esfahlani et al. (2020) and edge-centric functional connectivity proposed by Faskowitz et al. (2020) that was applied to human fMRI connectivity values. We integrate the information obtained from PLV and dPTE of the relevant associative network, quantifying the relationship between network edges to observe multipathway and frequency-specific motifs of informational processing and functional connectivity. We also observed their changes during the genesis of anxiety and investigated the relevance of such motifs in the alterations of behaviour of rats in an anxiogenic environment and the overall functional alterations of the network.

## MATERIALS AND METHODS

### Rats and Stereotaxic Surgery

Adult male Sprague-Dawley rats used in this study were bred and maintained by the Laboratory Animal Service Centre of The Chinese University of Hong Kong. The animal control room was controlled at a temperature of 23°C. All animals were handled in strict accordance with Chinese University of Hong Kong guidelines, and the procedures were approved by the Animal Experimentations and Ethics Committee. For surgical procedures, rats were anesthetized via intraperitoneal injection of ketamine (75 mg/kg, i.p.) and xylazine (6 mg/kg, i.p.) before being placed gently and fixed on the stereotaxic apparatus.

### 6-OHDA and Saline Infusion Into the DMS

A total of 27 male Sprague-Dawley rats with mean initial weight of around 260 g (each rat not exceeding 5 g from 260 g) underwent stereotaxic infusion of either 6-OHDA, in order to artificially deplete the dorsomedial striatum of its dopaminergic innervation, or infusion of saline for the control group. Cranial openings were made on the skull above the striatal region. Fourteen rats had 0.4 μl 6-OHDA of hydrobromide solution (40 μg 6-OHDA.HBr per 1 μl of sterile saline with 0.2% ascorbic acid) stereotaxically infused via a 1-μl Hamilton syringe into the anterior dorsomedial striatum (AP: 1.0 mm; ML: 2.3 mm; DV: 5.5 mm) at a rate of 0.06 μl/min. Thirteen rats received stereotaxic infusion of equal volumes of sterile saline at the same rate acting as the control group. The rats received subcutaneous anti-inflammatory, analgesic, and antibiotic injection immediately following the surgery. Rats were handled by the handler 5 min per day for 7 days after surgery.

### Behavioral Tests

Rats were first habituated to the behavioural room for 30 min before any test took place. The elevated plus maze was elevated 50 cm from the ground, with the closed arms the dimension of 15 × 50 cm (40-cm walls), the centre piece being 15 × 15 cm, the two open arms 15 × 50 cm. The behavioural room was lit via a 220-W fluorescent ring lamp. As rats explored the plus maze, AnyMaze software tracked the rats centre point on the maze and calculated various behavioural parameters.

### Electrode Implantation Into the Dorsomedial Striatum–Tied Associative Network Regions

Four rats treated with 6-OHDA and four rats treated with saline were allowed 1 week of recovery from the injection surgery and then unilateral implantation of electrodes (left side) were carried out. Electrodes constructed from twisting two strands of 0.00315-inch-diameter stablohm 675 wire (California Fine Wire Co.) into a 2- to 3-cm-long electrode; 250 μm of the recording tip of the electrode is deinsulated and 3 mm of the connector end of the electrode deinsulated and welded to adaptors. Cranial openings were made above the target brain regions; the electrodes were then lowered with the recording tips embedded into the target brain regions: mPFC (AP: 2.52 mm; ML: −0.34 mm; DV: 4.9 mm), ACC (AP: 1.08 mm; ML: −0.34 mm; DV: 2.5 mm), RSC (AP: −3.36 mm; ML: 0.34 mm; DV: 2.2 mm), CeA (AP: −2.76 mm; ML: −4.5 mm; DV: 8.7 mm), PtA (AP: −4.08 mm; ML: −3.0 mm; DV: 1.10 mm), and dHip (AP: −4.36 mm; ML: −1.6 mm; DV: 3.3 mm). The reference screw was screwed into the cerebrospinal fluid above the cerebellum and connected to the adaptor. Superglue and dental cement were used to stabilize the electrodes on the cranium. The adaptors were inserted into housing pieces and fixed onto the dental cement. Rats received subcutaneous anti-inflammatory, analgesic, and antibiotic injection immediately, 24 hours and 48 hours after the surgery. Rats were handled by the handler for 5 min per day for 10 days following surgery.

### Histology

Rats underwent transcardial perfusion of 4% PFA. Histological sectioning and analysis were performed after experiments were performed to confirm dopaminergic depletion of the DMS via staining of tyrosine hydroxylase (Primary goat anti-TH antibody, secondary anti-goat rabbit antibody) and to confirm correct placement of electrodes into the target brain regions.

### Local Field Potential Recording and Analysis of Local Field Potential Readings

Local field potentials (LFPs) were recorded from the target brain regions referenced against cerebellar activity with an acquisition rate of 1000 Hz. Ten days following electrode implantation rats were habituated in the recording room for 30 min before being connected to the Plexon electrophysiology acquisition system via a headstage and cable. Ten minutes of LFPs were recorded for rats when they were awake and resting in their home cage, 10 min when the rats were placed on the elevated plus maze to freely explore, and 10 min when the rats were immediately placed back to their home cage in the same room. Power spectra of local field potentials were calculated from the normalized raw 10-min voltage data notch filtered 0–0.01 Hz and 48–52 Hz. Relative power was calculated for each frequency from 0–100 Hz at 0.0244 Hz increments.

Theta oscillations were then isolated via band-pass filtering of 4–8 Hz oscillations from the raw LFP, and Hilbert transformation was applied to identify the phase between 0 and 2π of the signal at each recorded time point. Phase locking value was calculated between two LFP epochs of the same rat via the following equation:

$$PLV\left(X,Y\right)=\frac{1}{N}\sum_{n=1}^{N}e^{i\left(\theta_{x}\left(n\right)-\theta_{y}\left(n\right)\right)}$$

where *X* and *Y* are the LFP time series of two different brain regions, *N* is the number of data points of the LFP epoch (e.g., *N* = 1,000 for 1 s of LFP data), *θ*_*x*_(*n*) and *θ*_*y*_(*n*) is the phase of the oscillations of *X* and *Y* at that particular time point.

For phase transfer entropy (PTE), this involves the calculation of Shannon entropy or uncertainty of a particular stretch of time of LFP by observing the distribution of phases via the following equation:

$$H\left(\theta_{x}\left(t\right)\right)=\sum_{b=1}^{B}P\left(b\right)*\log\left(\frac{1}{P\left(b\right)}\right)$$

*H*(*θ*_*x*_(*t*)) is the Shannon phase entropy of a time series *X*, where *θ*_*x*_(*t*) is the phase of the oscillations of *X* for any time point *t*, *B* is the number of bins in which phase from 0 to 2*π* is divided, *b* is the numerical number assigned to a bin, with *P*(*b*) being the probability that at any time point the time series would be in a particular phase bin. The size of each bin was determined by the following equation first proposed by Scott (1992):

$$\text{Binsize}=3.49*\text{mean}\left(\sigma_{\text{phase}}\right)*N^{-1/3}$$

*N* is the number of data points within the LFP epoch, and *σ* is the standard deviation of signal phases within that instantaneous time. The phase of each data point is then fitted into bins created from dividing 0 to 2*π* into bin size intervals, where the number of bins is equal to 2*π*/bin size. The above equation is referenced from the “brainstorm-tools” code in Fraschini and Hillebrand (2016). Phase bin sizes of resting-state data (each segment and frequency band) are shown in Supporting Information Figure S1; there was a consistent bin number of 22 for the vast majority of segments in all frequency bands (only some delta band segments had deviations not exceeding four bins) and bin size of 0.22–0.31 for delta band and 0.28–0.30 rads for all other frequency bands in both saline and 6-OHDA treated rats.

As described by Lobier et al. (2014), in order to determine the information transfer between two LFP time series *X* and *Y*, with the phase *θ*_*x*_(*t*) and *θ*_*y*_(*t*), respectively, Shannon entropy of *Y*’s phase at the present timepoint *t* conditioned on its own past at time *t*′ is subtracted from the Shannon entropy of *Y*’s phase conditioned on its own past and also the past of *X*’s phase, as illustrated in the below equation:

$${PTE}_{X\to Y}=H\left(\left.\theta_{y}\left(t\right)\right|\theta_{y}\left(t^{\prime }\right)\right)-H\left(\left.\theta_{y}\left(t\right)\right|\theta_{y}\left(t^{\prime }\right),\theta_{x}\left(t^{\prime }\right)\right)$$

Conditional entropy was calculated by this general formula:

$$H\left(B|A\right)=-\sum_{a\in A,b\in B}p\left(a,b\right)\log\frac{p\left(a,b\right)}{p\left(a\right)}$$

Thus, the larger PTE is, the more *θ*_*x*_(*t*^′^) can reduce the entropy/uncertainty of *θ*_*y*_(*t*), suggesting a greater informational flow from the source origin of *X* to *Y*. The lag *δ* = *t* − *t*′, is determined by the number of full oscillation from 0 to 2*π* within a time series, thus, it is determined as:

$$\delta =\frac{N}{C}$$

*N* is the combined total data point from the two LFP time series compared, and *C* is the combined number of times with which the phase goes from less than *π* to being larger than *π*.

PTE value is then normalized over direction to give directed PTE as described by Engels et al. (2017):

$$\text{directed}\;{PTE}_{X\to Y}=\frac{{PTE}_{xy}}{{PTE}_{xy}+{PTE}_{yx}}$$

Thus, when directed PTE is larger than 0.5, there is preferential flow from *X* to *Y* and vice versa. In this manuscript, directed PTE value is further subjected to further adjustment by subtracting the directed PTE by 0.5

$$\text{directed}\;{PTE}_{X\to Y}=\text{directed}\;{PTE}_{X\to Y}-0.5$$

Thus, when dPTE is larger than 0, there is preferential flow and net outflow of information from *X* to *Y* and vice versa.

### A Framework of Edge-Based Network Analysis Based on Correlated Information Flow and Functional Connectivity

**Figure 1.**
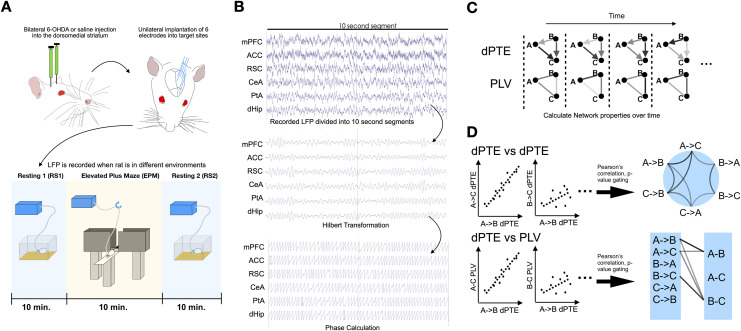
Workflow of edge-based network analysis of induced behavioral changes. (A) 6-OHDA or saline is infused into the dorsomedial striatum of Sprague-Dawley rats. Rats were then implanted with six electrodes and LFP recorded during the resting (RS), elevated plus maze (EPM), and resting 2 (RS2) state. (B) Due to the spontaneous and dynamic nature of informational flow and connectivity, LFP signals were divided into 10-s epochs, in which for each segment a band-pass filter is applied and phase calculated via Hilbert transformation. dPTE and PLV values were then calculated for each pair of brain regions for that particular LFP epoch. Epochs of LFP corresponding to rat transitioning were also isolated, and for each epoch a dPTE and PLV value were calculated. (C) Network properties dynamically change in a temporal dimension, with dPTE and PLV values fluctuating. (D) Network pathways that are strongly associated with each other can be unveiled by correlating dPTE values of each edge and direction and observing the extent of their covariation throughout time via Pearson’s correlation. In addition, information flow can be correlated with network organization via correlating dPTE with PLV values of various edges of the network to reveal pathways involved in network arrangement.

We propose a framework of network analysis based on the correlation of phase-based information flow and functional connectivity derived from LFP values. With respect to the Dorsomedial striatum–tied associative network (DSAN), following injection of 6-OHDA or saline into DMS, electrodes were implanted into target brain areas (Figure 1A). LFP were recorded from these areas when the animals underwent behavioural assessment in resting state before (RS), on an elevated plus maze (EPM), or post-EPM resting state (RS2) (Figure 1A). For each state, the LFP recording was first divided into fixed (10 s) epochs, which was then band-pass filtered through specific physiological frequencies (delta, theta, alpha, beta, gamma). Instantaneous phase of LFP oscillations were obtained after Hilbert transformation (Figure 1B). PLV and dPTE were then derived from the transformed LFP signals to quantify the functional connectivity and information flow in different times (Figure 1C). However, just comparing the differences between different states does not unveil much information regarding changes to the underlying dynamics of a network. As networks are dynamic and exhibit evolving interactions across time, especially during exploratory behaviour, the relationships between network parameters between different edges can reveal how different pathways across a network are associated with one another and whether bundles of pathways are regulated together. By correlating the dPTE values of each possible network edge direction against each other, a large-scale multipathway informational processing relationship of the network could be revealed (Figure 1D), in which changes in dPTE of individual pathways can be contextualized in a broader network organizational framework of dPTE changes in other pathways. In addition, by correlating dPTE values against PLV values of each brain pair and identifying significant, concurrent changes in informational flow and function (Figure 1D), we can unveil how functional connectivity within a network responds to information flow, which can give further insight into the self-regulation of networks, as was observed in approach/avoidance behaviours (Spielberg et al., 2012).

The use of 10-s epochs was a balance between having sufficient segments (around 60 per rat per state) for dynamic analysis and for each segment to have sufficient amount of phase changes for robust dPTE measurements, even for the delta band at 1–4 Hz (for 1 Hz, there will still be 10 full oscillations for that epoch with more than 20 phase bins). Fraschini et al. (2016) has recommended, based on human EEG data, using epochs of more than 6 s for more stable connectivity measurements, and their results showed that network parameters are more stable with epoch lengths approaching 10 s or above.

## RESULTS

### Increased Level of Anxiety in 6-OHDA-Treated Rats on the Elevated Plus Maze

**Figure 2.**
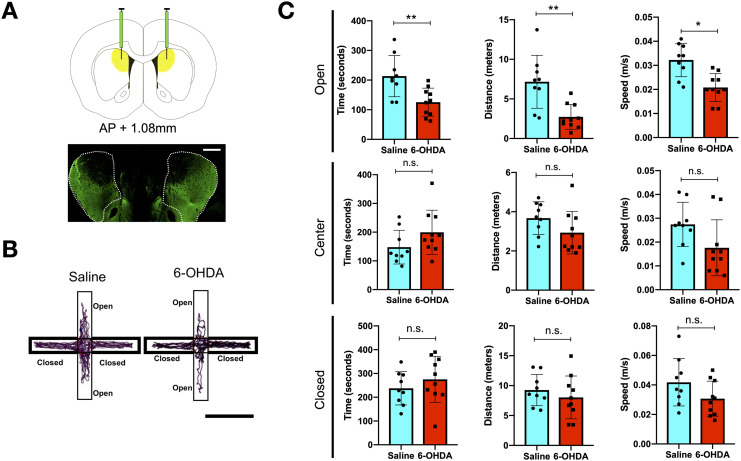
A 6-OHDA infusion and alterations to avoidance behaviors. (A) Schematic demonstrating the area in which 6-OHDA or saline will be infused into and representative TH staining of a 6-OHDA-infused rat, showing drastic depletion of dopaminergic fibres in the targeted dorsomedial striatum (bar = 1 mm). (B) Representative tracing of 6-OHDA- and saline-infused rat centre point on the elevated plus maze. (C) Time, distance, and speed of 6-OHDA- (*n* = 10) and saline-infused rats (*n* = 9) in the closed, centre, and open zones of the elevated plus maze. *p < 0.05, **p < 0.01 (two-sample *t* test).

Infusion of 6-OHDA into the DMS was performed to deplete dopaminergic fibres locally, which was then verified post-mortem via tyrosine hydroxylase (TH) staining (Figure 2A). Following 7 days of recovery and habituation to the experimenter, the rats were then placed on the EPM for 10 min of free exploration. Comparing the behavioral parameters with rats treated with saline (*n* = 9) to the DMS, 6-OHDA-treated rats (*n* = 10) have a significantly reduced time spent, total distance travelled, and average speed on the open arm of the EPM. On the EPM, 6-OHDA-treated rats have a significantly reduced time spent, total distance travelled, and average speed on the open arm. The differences were insignificant and less pronounced in the centre zone and completely negligible in the closed arm (Figure 2B and 2C). Open arms are considered to be more anxiogenic to rats due to a natural fear of heights and a tendency to stay in enclosed spaces (closed arm), and hence reduced spontaneous motor/exploratory behaviour as indicated by speed, distance travelled, and time spent on the open arm point toward increased anxiety (Walf & Frye, 2007). Such changes revealed an increase in anxiogenic avoidance behaviours by rats with focal dopaminergic depletion confined to the DMS.

### Changes in Posterior-Anterior Theta Band Power in an Anxiogenic Context

**Figure 3.**
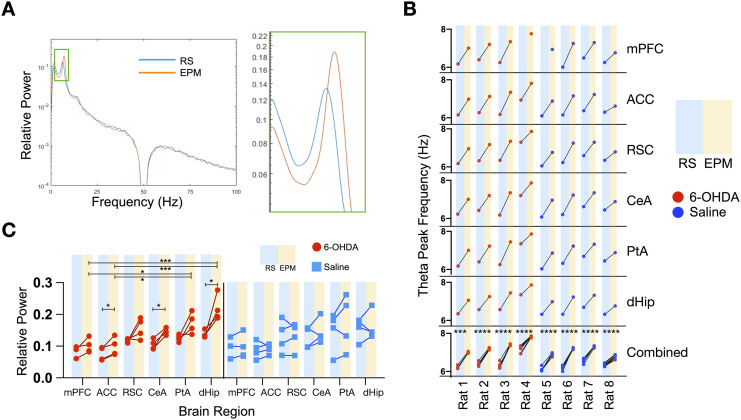
Theta band oscillatory changes in DSAN component brain regions. (A) Representative power spectra of control rat hippocampus during rest and EPM: Following z-transformation of 10-min LFP signals for each state, theta peak were present on almost all brain regions for all saline and 6-OHDA-infused rats during resting (one 6-OHDA-infused rat and one saline-infused rat do not possess a theta peak in the mPFC during the resting state) and shifts when placed on the EPM. (B) The frequency of the maximum relative power theta peak for each DSAN network brain region (*n* = 6 brain regions) for each individual rat in the resting state (RS) and on the EPM. (C) Relative theta peak power of each brain region in the resting state (RS) and on the EPM for 6-OHDA (*n* = 4) and saline-infused rats (*n* = 4). *p <0.05, **p < 0.01, ***p < 0.001, ****p < 0.0001 (paired *t* test).

Correct placement of recording electrodes targeting DSAN component regions in 6-OHDA or saline-treated rats was verified histologically post-mortem (Supporting Information Figure S2). Consistently, 6-OHDA-treated animals spent more time in both the transition zone and closed arm (Supporting Information Figure S3A, B), and transitioning into the closed arm for a higher number of times (Supporting Information Figure S3C). Similar changes related to transitioning were also observed in nonelectrode-implanted rats such as a tendency to enter a closed arm (Supporting Information Figure S4A), reduced time moving away from the closed arm (Supporting Information Figure S4B), and higher immobility in the centre zone (Supporting Information Figure S4C) in 6-OHDA-treated rats. Analysis of the power spectrum of the LFPs revealed a peak in the theta rhythm in most recorded brain regions in all the animals (Figure 3A). Notably, for both groups of animals, when comparing RS and EPM LFPs, there was a shift in theta peak frequency across all brain regions examined. Furthermore, in the same rat, the variation in the change of theta peak frequency among brain regions was very low (Figure 3B). These findings are consistent with theta rhythm being enhanced during exploratory behaviour. In contrast to the consistent shift in peak frequency, a differential response between control and 6-OHDA-treated rats was observed with respect to the changes in the relative power of the theta rhythm. For saline-treated rats, there were no significant difference (*p* = 0.1566, *F* = 1.835 one-way ANOVA test) in relative theta power between the brain regions and no significant changes in power when the rats were transferred to the EPM (Figure 3C). In contrast, in 6-OHDA-treated rats, we found that the relative power across individuals for each brain region exhibited less variation. Furthermore, for ACC, CeA, and dHip there was a significant increase in relative theta peak power when the rats were placed on the EPM. Also notable is that the theta power of posterior regions (PtA and dHip) on the EPM had a significantly higher value than the anterior regions (mPFC and ACC) (Figure 3C).

### Changes in Theta Band Informational Flow and Functional Connectivity in the DSAN

**Figure 4.**
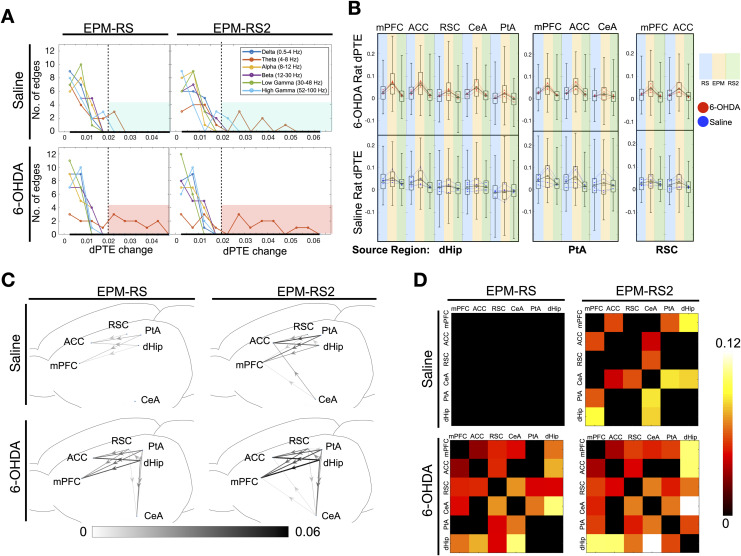
Changes in dPTE and PLV values from resting to EPM and from EPM to resting. (A) Histogram of dPTE scalar changes of network edges in various frequency bands when averaged dPTE values from EPM were subtracted with resting state (EPM-RS) and resting state 2 (EPM-RS2) values in 6-OHDA- and saline-infused rats (average of *n* = 240 epochs per state from four rats). (B) Bee swarm plot and box plot of theta band dPTE values of pooled individual epochs from the major bottom-up pathways with dHip, PtA, and RSC as the source region. Gray dot and lines indicate changes in group mean (average of *n* = 240 epochs per state from four rats), whereas red or blue dot and lines indicate changes in the mean of the epochs of each individual rat (60 epochs per state per individual rat) of 6-OHDA and saline-infused rats respectively. (C) Network schematic revealing the specific connections, directionality, and magnitude of the dPTE alterations in the theta band when averaged dPTE values from EPM were subtracted with resting-state (EPM-RS) and resting-state 2 (EPM-RS2) values in 6-OHDA- and saline-infused rats (average of *n* = 240 epochs per state from four rats) with a threshold of *p* < 0.001. (D) Matrices demonstrating difference in theta band PLV values across various the DSAN edges when rats were on the EPM and when in resting state (EPM-RS) and resting state 2 (EPM-RS2) (average of *n* = 240 epochs for each state from four rats).

We then asked whether there were frequency-specific alterations in information flow when the rats were in the EPM compared with the resting states. To do so, the averaged dPTE values of each frequency band on the EPM were subtracted with values obtained during RS and RS2. Consistent with a dominant role of theta activities, theta band alterations were the most widespread and prominent in terms of scale of change across the DSAN when the rats were on the EPM than during the resting states (Figure 4A) as seen by the higher number of network edges having a large scalar change in dPTE. If by examining the number of edges with a dPTE change of at least 0.02 (highlighted in red for 6-OHDA group, blue in saline group), for EPM-RS, saline group had a total of two edges and 6-OHDA rats had 10; for EPM-RS2, saline group had a total three edges and 6-OHDA rats had 10, showing that the theta dPTE increase was more prominent for 6-OHDA-treated rats (Figure 4A). When looking at each rat individually, we can see that 6-OHDA-treated rats when compared to saline-treated rats showed greater significance in theta dPTE changes in terms of the total number of edges, with *p* < 0.01 (Supporting Information Figure S5A, B)as well as overall *p* value of changes for each edge for EPM-RS and EPM-RS2 combined (Supporting Information Figure S5C). Examining the raw dPTE values for each LFP segment in each state revealed that, in 6-OHDA treated rats, dHip, PtA, and RSC outflow to the mPFC, ACC, and CeA exhibited a clear increase in dPTE values on the EPM and a decrease in RS2. In contrast, the theta band dPTE changes are much less consistent in the control animals (Figure 4B). When the magnitude of theta band dPTE change along with the directionality were plotted out, for 6-OHDA treated rats, the RSC-PtA-dHip region, that is, all the components of the posterior medial subnetwork, are the major theta outflow region with the PtA being the most prominent followed by the dHip (Figure 4C). In saline treated rats, there were less significant increases in the theta band dPTE of the same RSC-PtA-dHip outflow pathways (EPM-RS: 11 significant pathways in 6-OHDA group, 5 in saline; EPM-RS2: 12 in 6-OHDA, 7 in saline). The mean dPTE change of the outflow pathways were also lower as compared to the changes in 6-OHDA-treated rats (EPM-RS: 0.0240 for 6-OHDA group, 0.0090 for saline; EPM-RS2: 0.0306 for 6-OHDA, 0.0166 for saline). When placed back to their home cage in RS2, there is a corresponding decrease in theta band dPTE of roughly the same pathways for both 6-OHDA- and saline-treated rats.

For PLV, low-frequency bands (delta, theta, alpha) showed a greater increase in overall network connectivity, which was more prominent in 6-OHDA-treated rats (Supporting Information Figure S6). Looking at the theta band specifically, significant increases in PLV value between the CeA and RSC, PtA, dHip regions and also between the hippocampus and frontal regions regions were observed in 6-OHDA-treated rats (Figure 4D). Such prominent increases are not observed in saline-treated rats. Surrogate time series were generated via randomly shuffling LFP time segments of each channel by 1,000 times. The dPTE and PLV values among shuffled data and dPTE and PLV values approached zero, showing that the patterns of dPTE and PLV were not the consequence of random noise within the time series (Supporting Information Figure S7).

### Loss of Striatal Dopamine Alters Multiedge Theta Band Motifs

**Figure 5.**
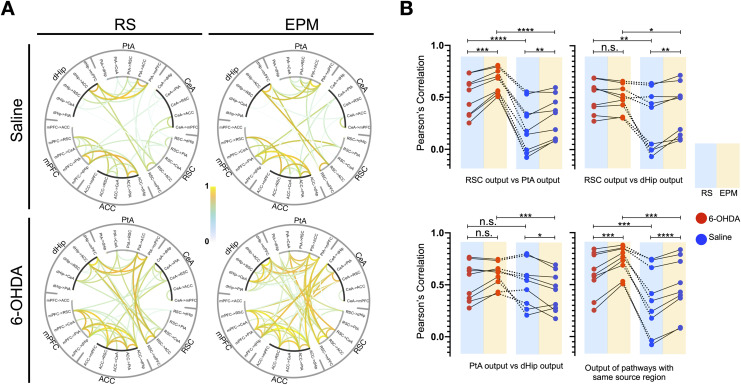
Altered theta band edge-based informational flow motifs in the DSAN following dopaminergic depletion. (A) Pearson’s correlation of theta band dPTE between all possible brain pairs/directionality reveals large-scale coregulatory informational outflow pathways (average of *n* = 240 epochs per state from four rats) in 6-OHDA- and saline-infused rats; correlations were presented at a threshold of *p* < 1 × 10^−10^ (Pearson’s correlation *p* value). (B) Differences between the correlation of RSC, PtA, and dHip outflow pathways to the CeA, ACC, and mPFC in 6-OHDA- and saline-infused rats during a resting and EPM state. Dashed lines indicate the same pathway combination compared in 6-OHDA and saline rats. Nine pairings between RSC and PtA outflow pathways, nine pathway pairings between RSC and dHip outflow pathways, and nine pathway pairings between PtA and dHip outflow pathways. Nine pairing between outflow pathways of the same source region. *p < 0.05, **p < 0.01, ***p < 0.001, ****p < 0.0001 (paired *t* test).

As pointed out previously, network configuration and metrics (i.e., dPTE, PLV) are not static and exhibit temporal fluctuations, transitioning through various network states. For example, we observed that the dPTE values of the bottom-up pathways in the DSAN fluctuate over time, with the fluctuations enhanced when the animals were on the plus maze (Supporting Information Figure S8), reflecting the spontaneous nature of brain networks. In order to pursue the question of the dynamics of theta band DSAN information flow, we took advantage of such fluctuations by looking into the large-scale multipathway informational processing relationship in the DSAN via correlating dPTE values of each possible edge direction against each other (example in Supporting Information Figure S9), gating for positive and significant correlations. When correlating dPTE values against each other, one can identify for 6-OHDA-treated rats significant correlation between dHip, PtA, and RSC outflow toward the mPFC, ACC, and CeA. Since the dPTE is antisymmetric and since Pearson’s correlation is nondirectional, reciprocal increases in correlation were also observed in mPFC, ACC, and CeA outflow toward the posterior regions (Figure 5A). However, since the correlation coefficients were enhanced when the animals were placed on the EPM and from the gross changes in theta dPTE there is an increased posterior to anterior flow, it is therefore more likely that dHip, PtA, and RSC outflow toward the mPFC, ACC, and CeA is responsible for the higher correlations observed.

To quantify the observed changes, correlation coefficients between RSC, PtA, and dHip outflow edges to the mPFC, ACC, and CeA pathways are compared (Figure 5B). In both saline- and 6-OHDA-treated rats, for correlations between pathways of the same source region (e.g., dHip outflow to mPFC vs. dHip outflow to CeA) and between outflow pathways of the RSC and PtA (e.g.. RSC outflow to the mPFC vs. PtA outflow to the mPFC), when the animals were on the EPM, correlation values increased. However, 6-OHDA-treated rats had higher correlation values in both resting and EPM state. For correlations between RSC and dHip outflow, 6-OHDA-treated rats had higher correlations in both resting and EPM state but only saline-treated rats exhibited an increase on the EPM. For correlations between PtA and dHip outflow, the EPM 6-OHDA-treated rats had higher correlations than saline-treated rats, but only saline-treated rats exhibited a decrease in correlation on the EPM.

### Correlation Between Informational Flow and PLV Corresponds With Network Changes During Transitioning

**Figure 6.**
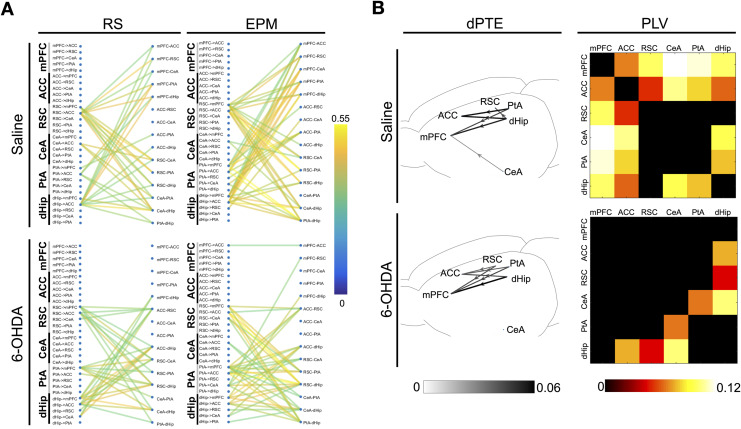
Theta band information flow-connectivity network regulatory motifs and theta band network response during transitioning. (A) Correlations between fluctuations in theta band dPTE with fluctuations in theta band functional connectivity, revealing functional organizational regulatory motifs across the DSAN (average of *n* = 240 epochs per state from four rats) in 6-OHDA- and saline-infused rats; correlations were thresholded at *p* < 1 × 10^−7^ (Pearson’s correlation *p* value). (B) Network schematic unveiling the specific connections, directionality, and magnitude of the dPTE alterations in the theta band when averaged dPTE values from transitioning (*n* = 77 epochs from four saline-infused rats; *n* = 75 epochs from 4 6-OHDA-infused rats) subtracted from averaged EPM dPTE values (Transition-EPM) (average of *n* = 240 epochs per state from four rats) with a threshold of *p* < 0.01 (two-sample *t* test) and matrices comparing differences in theta band PLV of transitioning epochs (average of *n* = 77 epochs from four saline-infused rats; *n* = 75 epochs from 4 6-OHDA-infused rats) versus the average PLV values on the EPM (Transition-EPM) (average of *n* = 240 epochs for each state from four rats) in 6-OHDA and saline-infused rats.

The relationship between informational flow and network connectivity is often overlooked in network analysis, which neglects the fact that nodes of a neural network do not just merely connect with one another, but also projects information. By correlating theta dPTE and PLV, we can understand the dynamics underlying theta band informational transmission and its influence on network organization. In saline-treated rats, RSC, PtA, and dHip theta outflow to the mPFC exhibits particularly strong correlations with mPFC theta band connectivity to the rest of the network, which was further enhanced when the animals were placed on the EPM (Figure 6A). This indicates that bottom-up informational flow is correlated with the centrality of the mPFC in the network. For 6-OHDA-treated rats, there is a dramatic reduction in the correlation between mPFC theta band connectivity with the rest of the network and RSC, PtA, and dHip theta band outflow to the mPFC. The results correspond to network changes in transitioning between maze arms, which is defined as the period of time starting when the head of the rat begins to exit an arm and ends when the base of tail along with the entire body enters another arm. During transitioning epochs, dPTE changes are also most prominent in the theta band (Supporting Information Figure S10). As shown in Figure 6B, 6-OHDA- and saline-treated rats have similar dPTE increases from the dHip and PtA to the mPFC and ACC. The only difference here is that for saline-treated rats, RSC did not have significant increase in informational flow toward the mPFC and ACC and even has net input from the dorsal hippocampus during transitioning. For functional connectivity, during transitioning, frontal regions in particular with the mPFC connectivity with the entire network, especially the PtA and CeA, are enhanced in saline-treated rats; the centrality of the network thus shifts toward the frontal regions. Such changes are absent for 6-OHDA-treated rats.

## DISCUSSION

In this study, we interrogated the changes in DMS-regulated associative network parameters in response to anxiogenic environments in both focal dopamine-depleted and control rats via multisite LFP recordings. As the functional divisions of the striatum and other brain network architecture are largely conserved across mammals, it is reasonable to assume that anxiogenic processing takes place through an evolutionarily conserved network mechanism (Adhikari, 2014; Kovner et al., 2019; Nikolova et al., 2018). We extended the utilization of the phase-based metrics, dPTE and PLV, in tracking concomitant functional and informational flow relationship across multiple network edges, allowing for larger emerging network patterns to be unveiled. Our study explored the possible role of dopaminergic innervation in the DMS in regulating anxiogenic behaviours in a novel environment, where artificial depletion via 6-OHDA leads to an increase in avoidance behaviours and decrease in exploratory drive in an anxiogenic environment. With the hippocampus being a major generator of theta oscillations and being highly responsive to exploratory behaviour (Buzsáki, 2004; Ekstrom et al., 2005; Whitlock et al., 2008), the major focus of this study was, when compared with saline-treated rats, whether 6-OHDA-treated rats demonstrated abnormalities in terms of theta band power and network organization changes between rest and when on the EPM.

First, we showed that there was a consistent network-wide increase in theta peak frequency on the EPM in both groups. However, the loss of dopamine in DMS leads to a differential network-wide theta power organization and response, with theta power showing lower variation, more consistent increases in power on the EPM, and is exaggerated in the posterior hippocampal and parietal regions. Based on analysis of dPTE and PLV measures, the three brain regions of the posterior medial subnetwork (Cwik et al., 2014; Ritchey & Cooper, 2020), that is, RSC, PtA, and dHip, exhibit exaggerated theta band outflow when 6-OHDA-treated rats were transferred to the EPM. The main recipients of these outflows were the mPFC and ACC and the CeA. The parietal and hippocampal regions were most dominant in the subnetwork and showed most significant outflow to the amygdala. Theta band functional connectivity following 6-OHDA infusion further showed that CeA increased its connectivity with the posterior medial subnetwork, suggesting the amygdala was more functionally incorporated into the subnetwork. We can hereby see an obvious posterior-anterior divide in terms of both theta power and theta information flow that was exaggerated under dopaminergic depletion. The predominance of the theta band in the posterior cortical regions is a well established phenomenon in rats, where the coupling of the hippocampus and posterior midline cortical structures and neurons, especially during exploration, had been demonstrated in multiple studies (Colom et al., 1988; Young & McNaughton, 2009). The posterior medial subnetwork has been well characterized to be involved in sensory information processing such as navigation, memory, and vividness of emotion representation (Cwik et al., 2014; Ritchey & Cooper, 2020), and the more anteriorly located mPFC and ACC are higher ordered affective and cognitive centres. We speculate in 6-OHDA-infused rats, due to a reduction in frontal regulation, there was an increase in bottom-up information transfer from the posterior medial subnetwork with a greater sensory processing role to the higher ordered frontal mPFC/ACC regions and the amygdala. From the results, we postulate that dopaminergic signalling of the dorsomedial striatum contributes to long-distance top-down frontal to posterior medial subnetwork mediation of informational flow. This may allow for higher ordered frontal regions to effectively regulate contextual and spatial information to prevent an excessive anxiogenic response from environmental stimuli. This notion relates with human studies where frontal midline theta rhythm has been found to be weakened in PD patients (Singh et al., 2018).

Brain networks, in particular, associative cortical networks, can be characterized not only in the spatial but also in the temporal domain (Pedersen et al., 2017; Vidaurre et al., 2017). In this study, the focus on the correlation across multiple network edges enabled us to further understand the aberrant theta outflow of the posterior medial subnetwork and identify abnormal network-wide theta band processing and network regulatory motifs. From dPTE-dPTE correlations, compared with saline-treated rats, 6-OHDA-treated rats had a hypersynchrony (general increase in correlation) between posterior medial subnetwork outflow pathways even during resting states that was further exaggerated in anxiogenic environments, forming a single dominant outflow cluster. One possible interpretation is that in addition to an excessive posterior medial subnetwork information outflow, dopaminergic depletion of the DMS may change the subnetwork from providing differentiated and orderly information to becoming more chaotic and unregulated. Thus, dopaminergic innervation to the DMS may be the key in delineating different pathway bundles to ensure well-regulated and diverse informational content across the DSAN.

From the results of correlating dPTE and PLV, in saline-treated rats, the posterior medial subnetwork outflow to the frontal regions is key to mPFC centrality, which was enhanced in an anxiogenic environment. The results seem to correspond well with theta band network processing during transitioning between maze arms. Saline-treated rats exhibited increased bottom-up theta band outflow and showed strong increase in mPFC connectivity with the other components of the DSAN. However, for 6-OHDA treated rats, bottom-up outflow to the frontal regions was also enhanced but at the same time showed an absence of increased mPFC connectivity and centrality within the entire DSAN, which corresponded to the loss of correlation between bottom-up outflow and mPFC centrality. This points toward the importance of frontal theta engagement in response to bottom-up outflow in initiating transitions to an open arm. As transitioning is a process where an integration of contextual information and internal anxiety bridges with decision-making, a robust frontal theta engagement with the network may be necessary to suppress the intrinsic anxiogenic responses to contextual information and generate the motivation to engage in exploratory behaviour. The loss of frontal network engagement therefore may lead to dysregulation and hypersynchrony of bottom-up pathways as seen in dPTE-dPTE correlations and hence an exaggerated context-dependent anxiogenic response.

Previous studies have shown that especially band-passed LFP time series are autocorrelated across time (Afyouni et al., 2019), which can lead to inflated connectivity values due to undersampling of state space. However, as our main results are not derived from the raw PLV and dPTE values but from changes in connectivity values across different conditions and correlating the fluctuations of said connectivity values against each other, this effect is unlikely to be a major confounder of the conclusions of this study. Nonetheless, given the relatively small sample size in this study, the conclusion obtained here warrants further investigation for confirmation and extension.

In conclusion, by integrating edge-based frequency-specific network dynamics with gross network and regional changes, we showed that a dorsomedial striatal-tied associative network in rats may be associated with the regulation of exploratory and avoidance behaviours in an anxiogenic context via theta band–mediated information transfer. This suggests that behavioural changes associated with anxiety may arise from degenerative neural disorders via spatial and temporal changes in network signalling. This approach can be further applied to other cognitive disease models to unearth the principles behind pathological behavioural alterations and network regulatory mechanisms.

## SUPPORTING INFORMATION

Supporting information for this article is available at https://doi.org/10.1162/netn_a_00251.

## AUTHOR CONTRIBUTIONS

Yin-Shing Lam: Conceptualization; Data curation; Formal analysis; Investigation; Methodology; Visualization; Writing – original draft; Writing – review & editing. Xiu-Xiu Liu: Formal analysis; Methodology. Ya Ke: Conceptualization; Investigation; Methodology; Project administration; Supervision; Writing – review & editing. Wing-Ho Yung: Conceptualization; Investigation; Methodology; Project administration; Supervision; Writing – review & editing.

## FUNDING INFORMATION

Wing-Ho Yung, Hong Kong Research Grants Council Theme-Based Research Scheme (T13-605/18-W). Ya Ke, Hong Kong Health and Medical Research Fund (07180906).

## TECHNICAL TERMS

Network edges: A measure of association or interaction between two brain regions or nodes of a broader network.
Phase locking value: A measure of functional connectivity between two brain regions by measuring if there is a consistent phase difference between the oscillatory local field potential of the two regions.
Phase transfer entropy: A measure of directional information flow via Shannon entropy of phase distribution between oscillatory local field potential of two brain regions.
Theta band: Oscillations of local field potentials at a frequency of 4 to 8 Hz.
Posterior medial subnetwork: A group of associative regions located posteriorly of the brain, encompassing the retrosplenial, parietal associative cortices and dorsal hippocampus that have close functional ties.
6-Hydroxydopamine: A neurotoxin that specifically targets and depletes dopaminergic and noradrenergic neurons.
Elevated plus maze: An animal behavioral test that measures level of innate anxiety by measuring time, speed and distance travelled on either the open arm, closed arm (with walls) or the center piece connecting the arms of a plus-shaped maze elevated from the ground.
Dorsomedial striatum–tied associative network: A collection of cortical and subcortical structures that have been demonstrated to have direct neural connections with the dorsomedial striatum.
Edge-based network analysis: Analysis of networks with the emphasis on edge properties and interactions between edges.
Network regulatory motifs: Higher order associations between edges as measured via co-fluctuations in dynamic information flow and functional connectivity.
Network dynamics: The characterization of fluctuations in network properties, in particular edge properties or node properties, temporally.
