# Supplementary material for: Edge-based network analysis reveals frequency-specific network dynamics in aberrant anxiogenic processing in rats
Source: Netw Neurosci. 2022 Jul 1;6(3):816–33. doi: 10.1162/netn_a_00251 (PMC9810363; doi:10.1162/netn_a_00251)
Supplement: Supplementary file 1 [file netn-06-816-s001.pdf]

# Supplementary Figure 1

Lam, Y., Liu, X., Ke, Y. & Yung, W. (2022). Supporting information for "Edge-based network analysis reveals frequency specific network dynamics in aberrant anxiogenic processing in rats." *Network Neuroscience*, *6*(3), 816–833. https://doi.org/10.1162/netn_a_00251

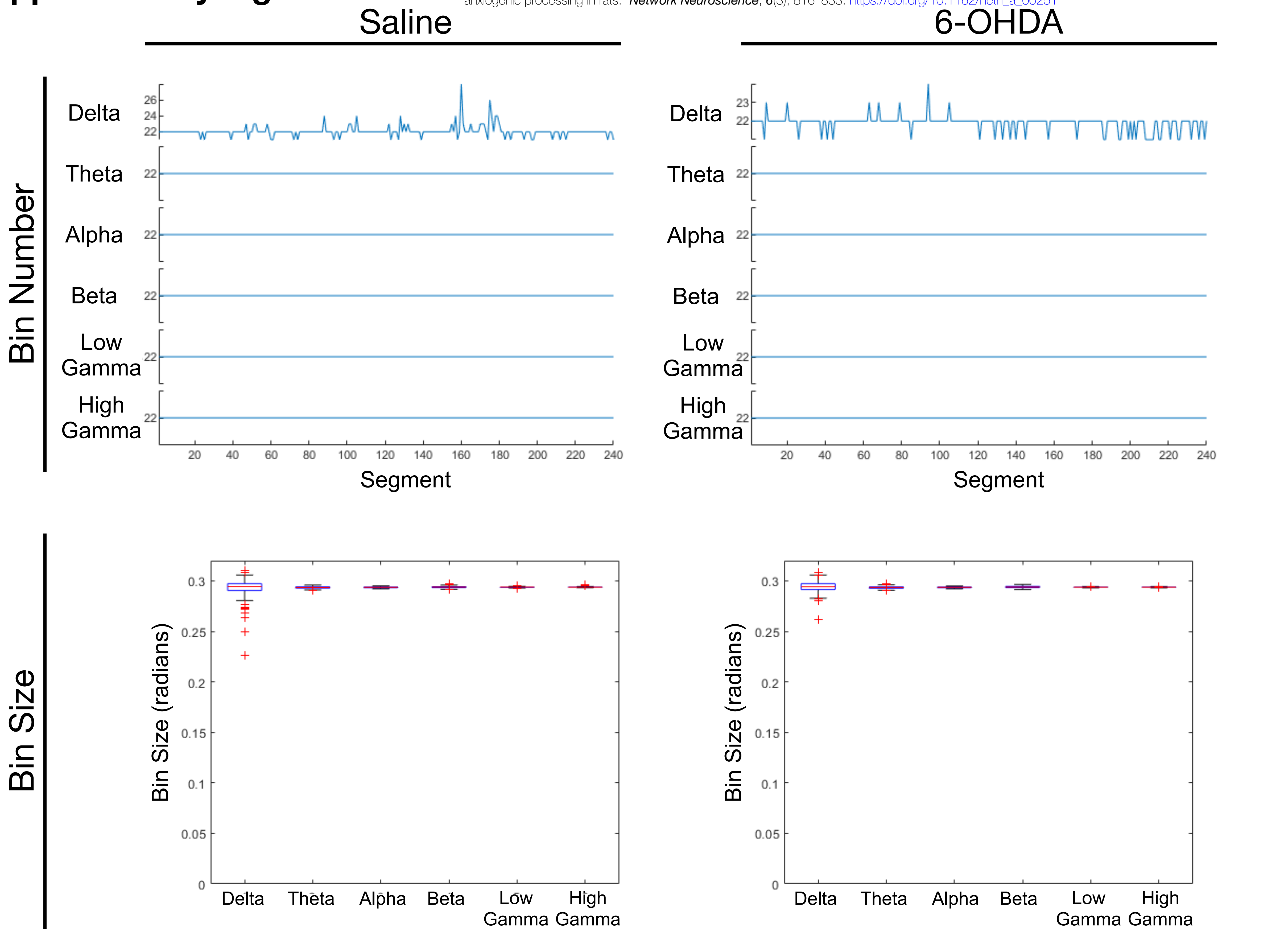


**Figure S1. Bin number and bin size for dPTE calculation** Bin number and bin sizes used in dPTE calculations for all frequency bands in time segments for saline treated and 6-OHDA treated rats at rest (240 epochs from 4 rats).

# Supplementary Figure 2

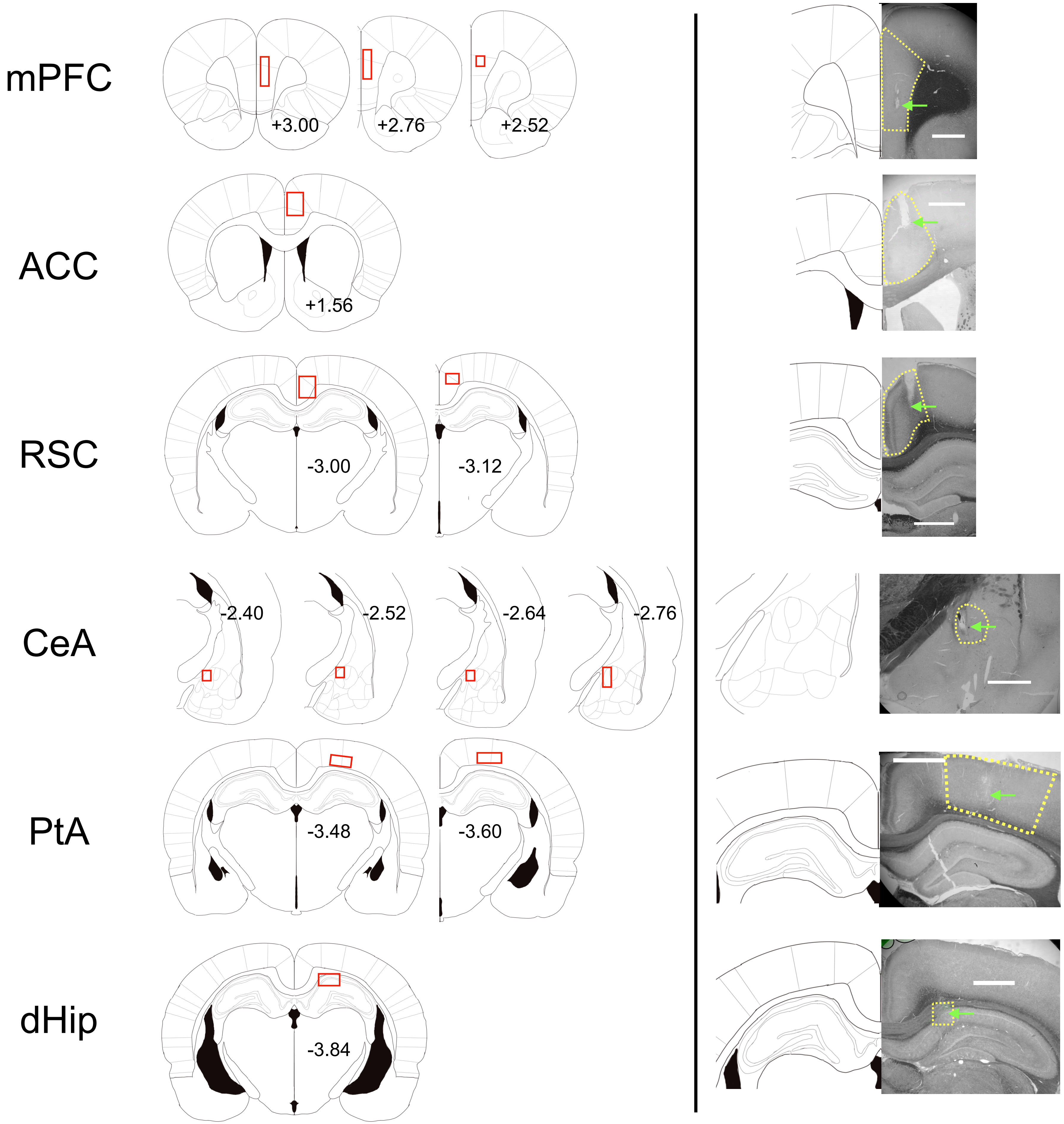


**Figure S2. Verification of electrode placement.** Schematics outlining the range of locations of the recording electrode tips, with representative photomicrographs showing on the right. The yellow boxes indicate the target regions and the green arrows indicate the identified electrode tips. Scale bar = 1mm.

# Supplementary Figure 3

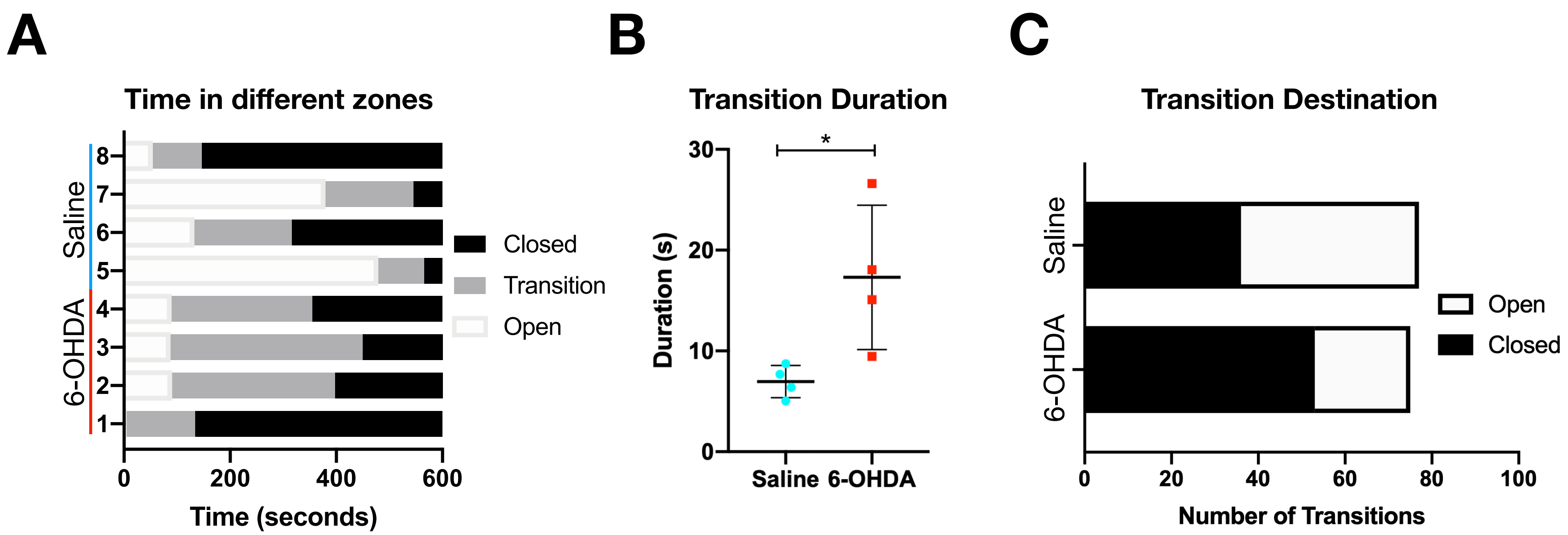


**Figure S3. Behavioral properties of electrode implanted rats. (A)** Time spent in different zones/ transitioning between 6-OHDA and saline infused rats. **(B)** Average duration of one transition for 6-OHDA and saline infused rats *p<0.05 (Mann Whitney U test). **(C)** Comparison between the number and type of transition in 6-OHDA and saline rats.

## Supplementary Figure 4

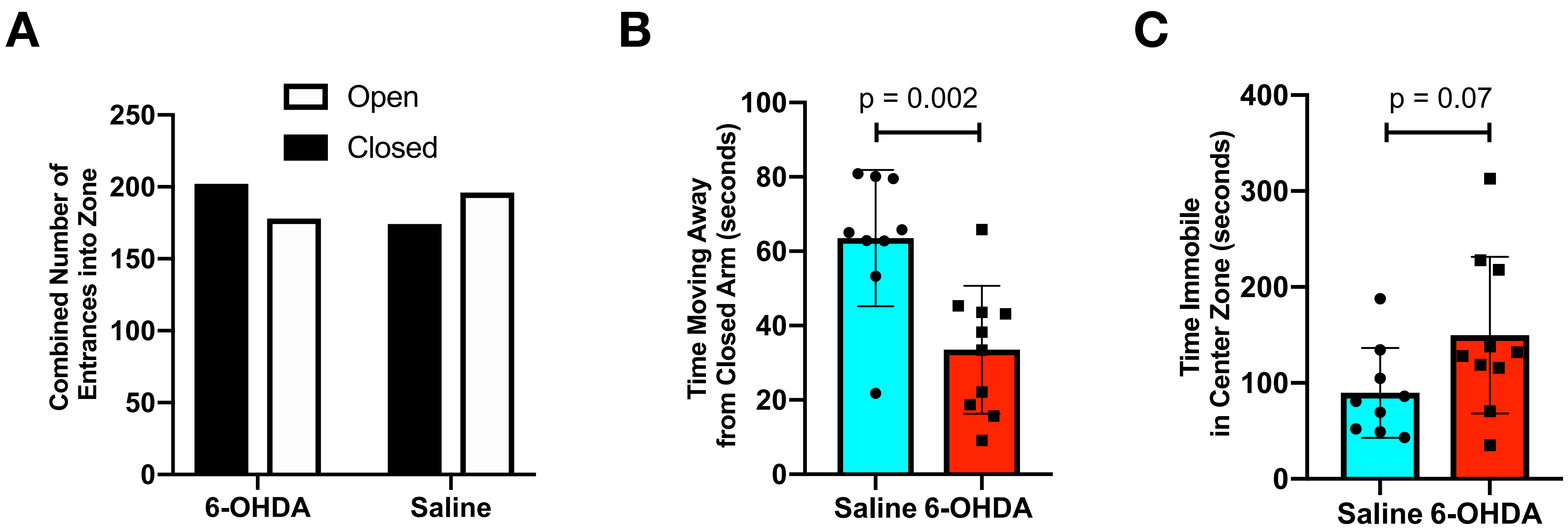


**Figure S4. Transition properties of non-electrode implanted rats via center point tracking (A)** Comparison between total combined number of entrances to the open or closed arm for 6-OHDA (n = 10) and saline treated (n = 9) rats **(B)** Time moving away from closed arm and time immobile at center zone in 6-OHDA (n = 10) and saline treated (n = 9) rats. **(C)** Time spent immobile in center zone in 6-OHDA (n = 10) and saline treated (n = 9) rats.

# Supplementary Figure 5

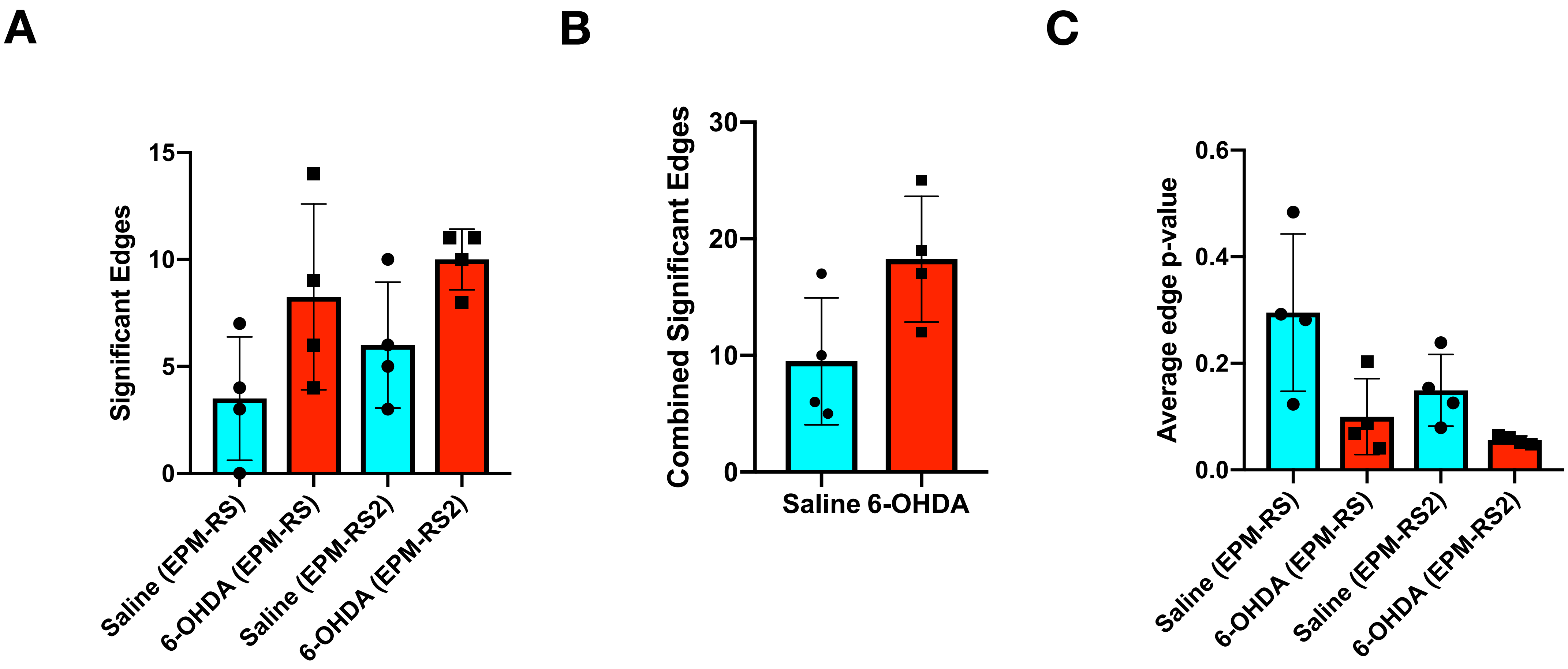


**Figure S5. Significance of theta band dPTE changes (A)** Number of edges with theta dPTE difference significance of $p < 0.01$ between EPM-RS and EPM-RS2 (60 segments per rat per state) in each 6-OHDA (n = 4) and saline rats (n= 4). **(B)** Combined number of edges with theta dPTE difference significance of $p < 0.01$ for EPM-RS and EPM-RS2 (60 segments per rat per state) in 6-OHDA (n = 4) and saline (n = 4) rats. **(C)** Average p-value of theta dPTE difference for all edges for EPM-RS and EPM-RS2 (60 segments per rat per state) in 6-OHDA (n = 4) and saline (n = 4) rats.

# Supplementary Figure 6

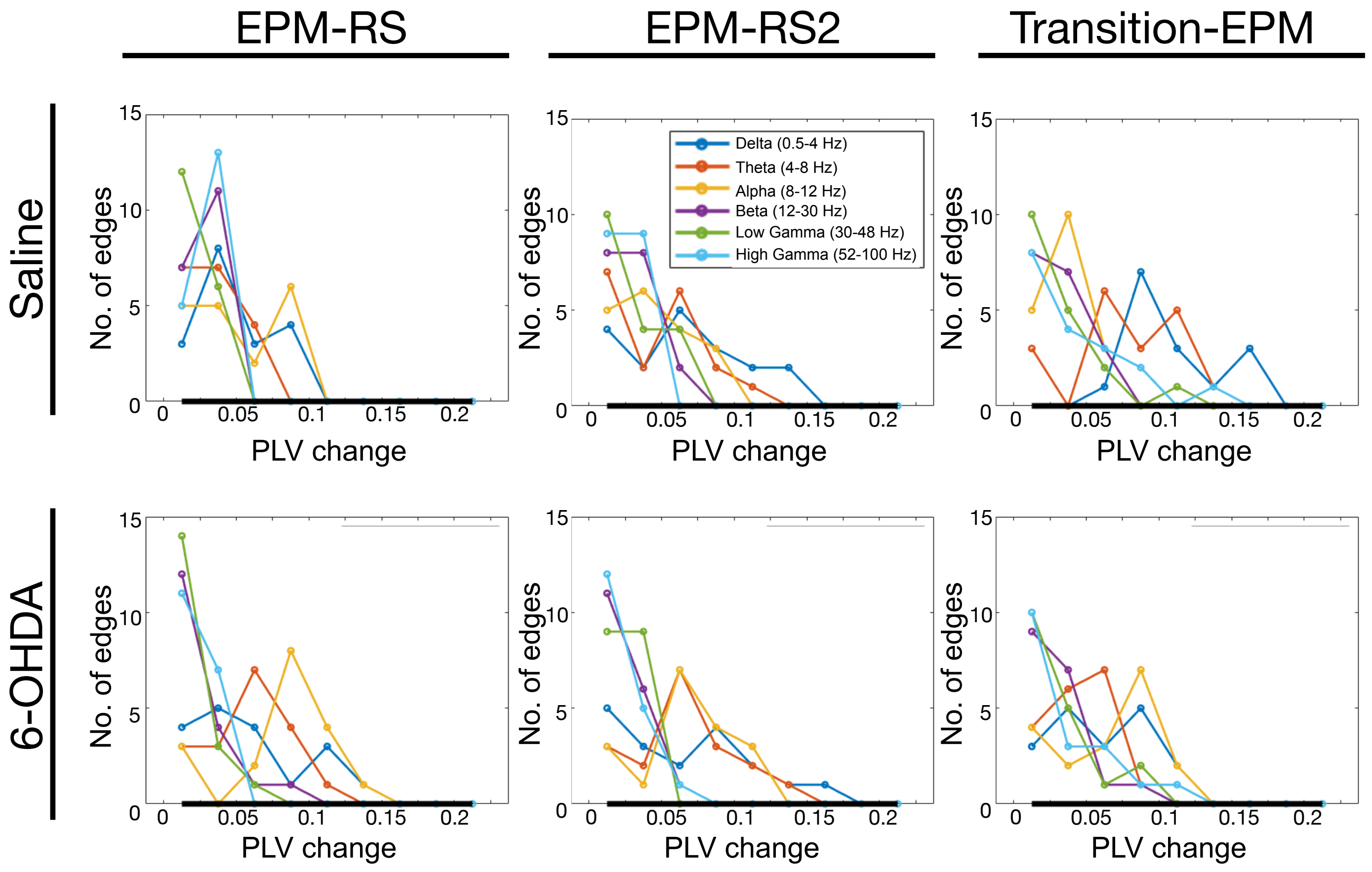


**Figure S6. PLV scalar changes during transitioning epochs.** Histogram of scalar changes in PLV across edges of the DSAN across various frequency bands when averaged dPTE values from EPM, were subtracted with resting state (EPM-RS) and resting state 2 (EPM-RS2) values in 6-OHDA and saline infused rats (n = 240 epochs per state from 4 rats).

# Supplementary Figure 7

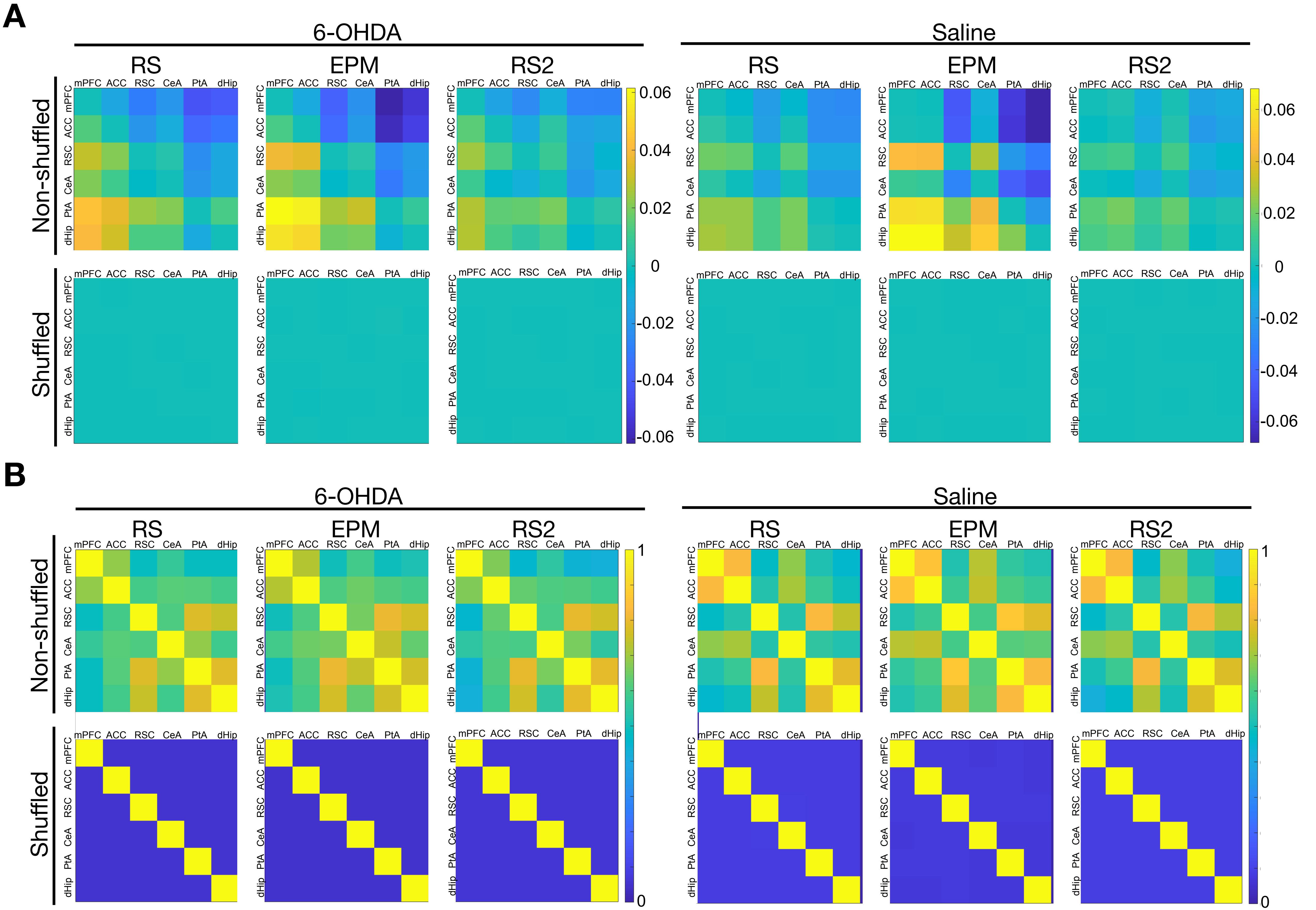


**Figure S7. Patterns of dPTE and PLV were not the consequence of random noise.** Matrices showing the average theta band dPTE and PLV value (n= 240 segment per state from 4 rats) at each state for 6-OHDA and saline rats and the corresponding dPTE and PLV values after the LFP time series has been shuffled randomly for 1000 times.

# Supplementary Figure 8

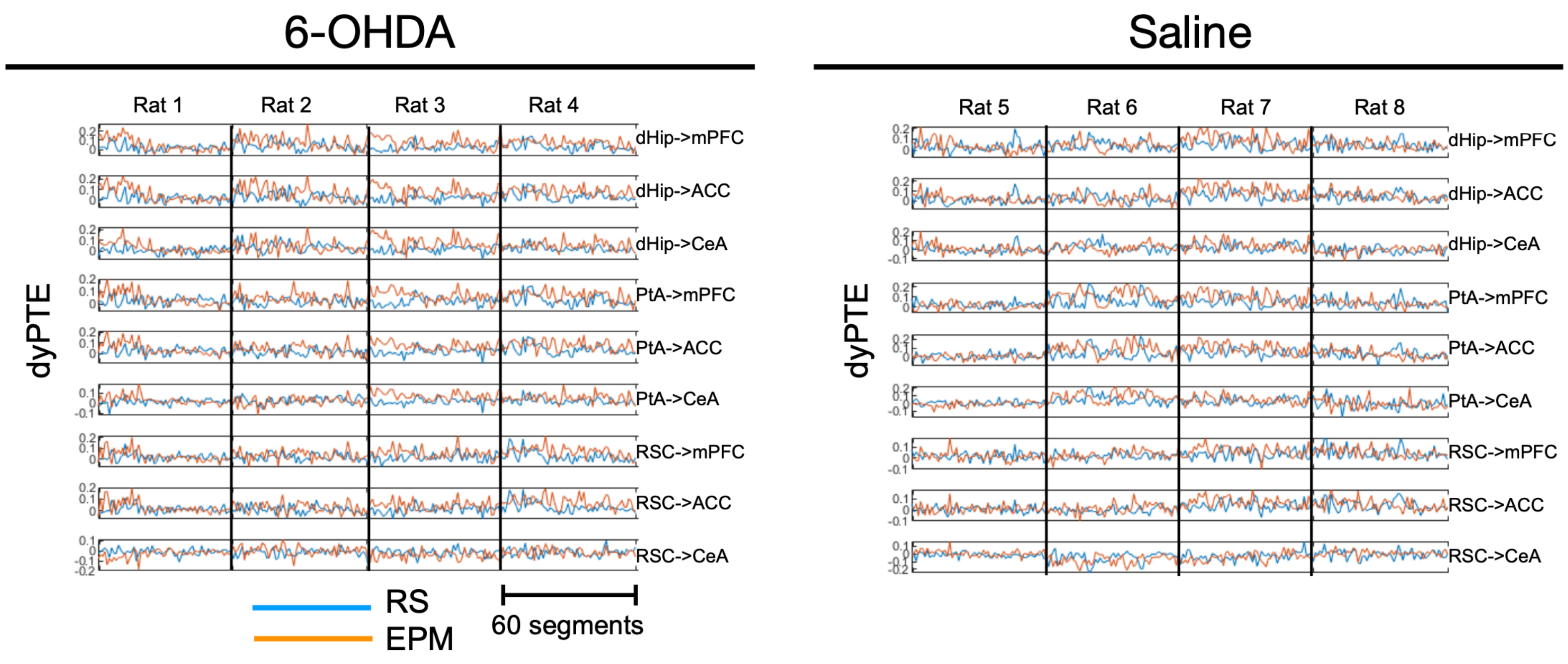


**Figure S8. Fluctuations of dPTE values in resting states and EPM.** On the right, bottom-up pathways in 6-OHDA and saline infused rats both demonstrate flux in dPTE values in both resting state and on the EPM with certain pairs of brain regions having a similar temporal profile of dPTE flux

# Supplementary Figure 9

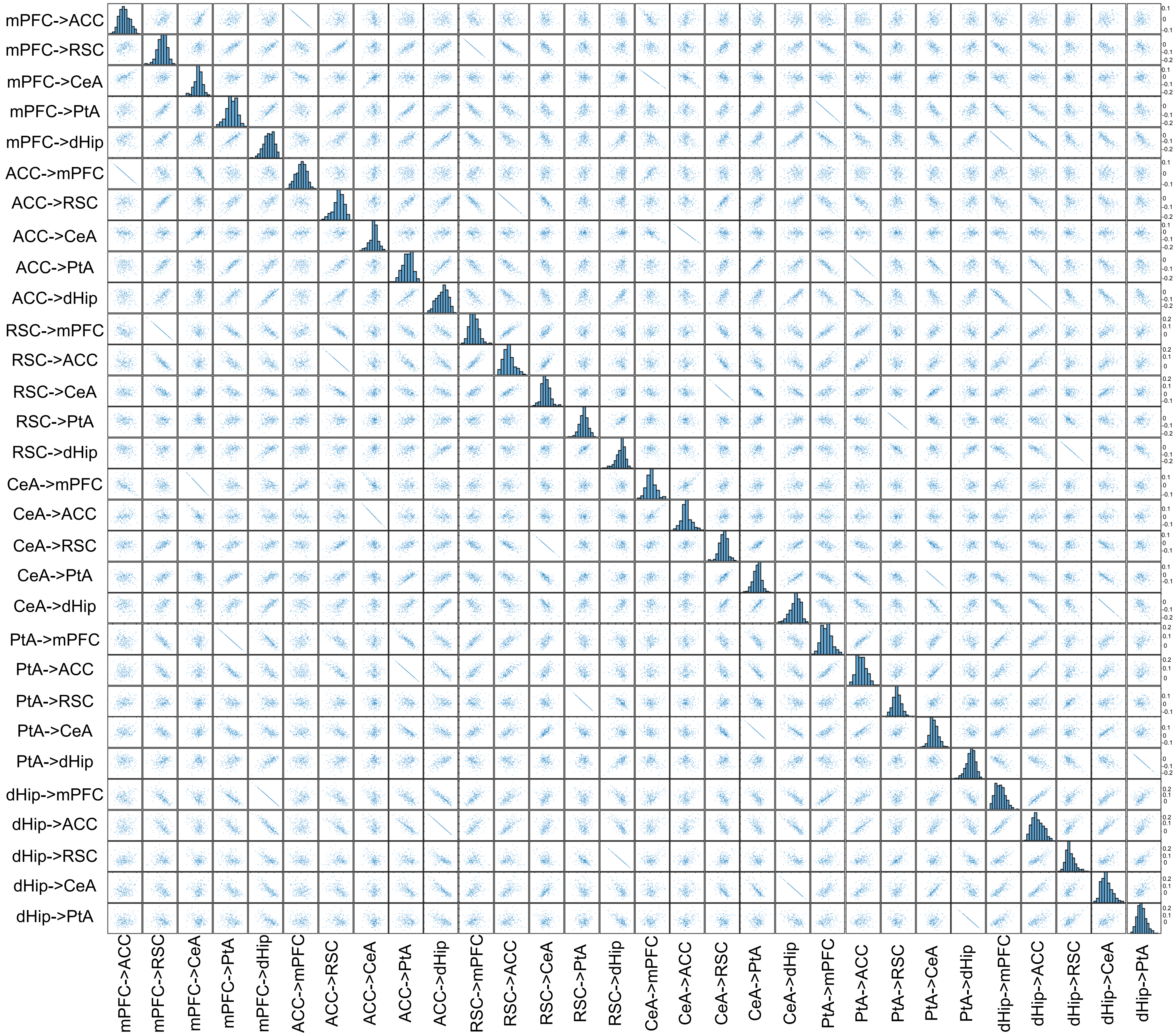


**Figure S9. Correlations of dPTE values of each possible edge direction against each other.** Correlation plots of dPTE values of each segment (n = 240 segments from 4 rats) between all possible combinations of directed edges of the DSAN in 6-OHDA treated rats on the EPM.

# Supplementary Figure 10

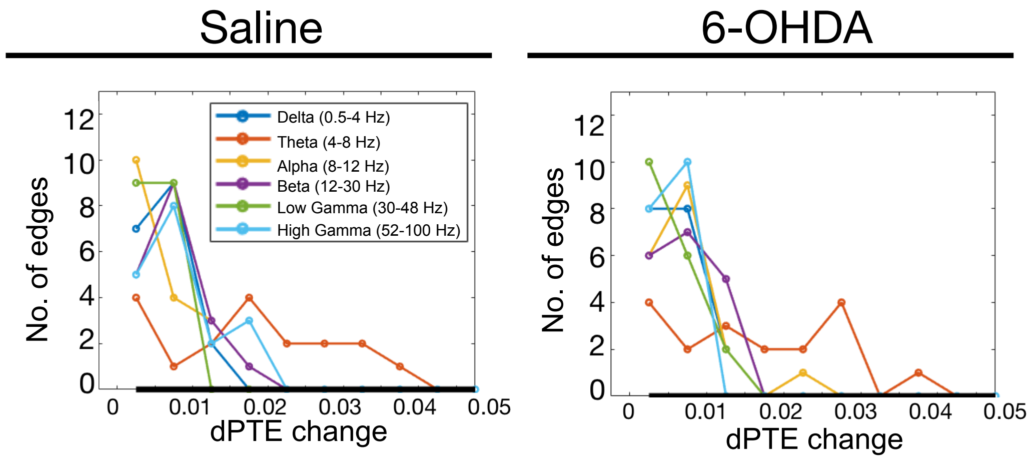


**Figure S10. dPTE scalar changes from transitioning.** Histogram of dPTE scalar changes of network edges in various frequency bands when averaged dPTE values from transitioning (n = 77 epochs from 4 saline infused rats, n = 75 epochs from 4 6-OHDA infused rats) subtracted from averaged EPM dPTE values (Transition-EPM) (n = 240 epochs per state from 4 rats).
